# Study of pulmonary involvement in HIV-seropositive patients

**DOI:** 10.1186/1471-2334-12-S1-P4

**Published:** 2012-05-04

**Authors:** Girija Nair, Hafiz Deshmukh, TK Jayalakshmi, Abhay Uppe, Kavita Modi

**Affiliations:** 1 Padmashree Dr D Y Patil Medical College and Research Center, Navi Mumbai, Maharashtra, India

## Background

HIV causes diseases of respiratory system in various ways. The lung involvement may be of infective etiology or non-infective like Kaposi’s sarcoma or pulmonary hypertension. In India with the load of pulmonary tuberculosis, immuno-compromised patients are exposed to the risk of tuberculosis. This study endeavors to identify the patients who get lung involvement secondary to HIV infection and impact of CD4 cell count on the severity of tuberculosis in HIV positive patients.

## Methodology

Observational cross sectional study of 50 HIV seropositive patients with pulmonary involvement attending OPD and admitted in Pad. Dr D Y Patil Medical College and Research Centre, Mumbai.

## Results

50 HIV patients were studied for 2 years. Majority of patients were in the age group of 40-49 years. 68% were males and 32% females. 62% patients had pulmonary TB. 28% had pleural effusion, PCP infection 2%, and pulmonary hypertension in 8%. Infiltrative lesions (42%) were more common X-ray findings. Sputum AFB positivity was seen in 52%. Mean CD4 count being 163.7cells/µl. Most patients (70%) had CD4 count<200 cells/µl. Mean CD4 counts in patients with sputum positive TB was 183.8cells/µl, in extra pulmonary tuberculosis was 174.9cells/µl, in sputum negative TB was 93.8cells/µl and in miliary TB it was 77.7cells/µl.

## Conclusion

There can be variety of lung involvement in HIV positive patients who can have many pulmonary infection as well as pulmonary hypertension, which may be a major cause of morbidity and mortality in HIV positive patients. CD4 counts were very low in military TB and sputum negative pulmonary TB.

